# Conservative Treatment of HAV‐Associated Acalculous Acute Cholecystitis in an Adult: A Case Report

**DOI:** 10.1155/carm/3446365

**Published:** 2025-12-11

**Authors:** Sebastian A. Medina-Ramirez, Camila A. Arones-Santayana, Eduardo Carcausto Huamani, Nelson Diaz-Reyes

**Affiliations:** ^1^ P53 Research Group, Human Medicine School, Peruvian Union University (UPeU), Lima, Peru; ^2^ Center for Research in Primary Health Care, Universidad Peruana Cayetano Heredia, Lima, Peru, upch.edu.pe; ^3^ International Clinic, Lima, Peru; ^4^ School of Medicine, Peruvian Union University, Lima, Peru; ^5^ Department of Internal Medicine, Good Hope Clinic, Lima, Peru

**Keywords:** acalculous cholecystitis, cholecystitis, conservative therapy, hepatitis A

## Abstract

Hepatitis A virus (HAV) is a viral infection that can present with a wide range of clinical manifestations, including rare complications such as acute acalculous cholecystitis (AAC). We present the case of a 31‐year‐old woman who arrived at the emergency department with severe epigastric pain, fever, early satiety, nausea, and vomiting. Physical examination revealed a positive Murphy’s sign, hepatomegaly, and epigastric tenderness, suggesting obstructive acute cholecystitis. Imaging studies showed reactive changes in the gallbladder and mild dilation of the intrahepatic bile ducts. Laboratory tests indicated elevated bilirubin levels and a cholestatic pattern with markedly increased transaminases. Magnetic resonance cholangiopancreatography confirmed AAC and ruled out biliary obstruction. Positive serology for Hepatitis A led to the definitive diagnosis of AAC secondary to Hepatitis A. Treatment focused on supportive care with intravenous hydration and symptomatic management, avoiding antibiotics due to the self‐limiting nature of the viral infection. The patient showed a favorable clinical course, with a progressive decrease in gallbladder diameter and normalization of liver parameters. This case highlights the importance of considering rare viral infections as a cause of AAC and demonstrates that a conservative approach can be effective in managing this condition. This case underscores the importance of considering viral etiologies, particularly HAV, in the differential diagnosis of AAC in adults. It further demonstrates that, in carefully selected patients, conservative management can be both safe and effective, thereby avoiding unnecessary antibiotic use or surgical intervention in the context of a self‐limiting viral infection.

## 1. Introduction

Hepatitis A virus (HAV) is a single‐stranded RNA virus belonging to the Hepatovirus genus of the *Picornaviridae* family [1]. It is primarily transmitted via the fecal–oral route, spreading directly from an infected person to a healthy individual or through the consumption of contaminated food or water [1, 2]. In 2019, the World Health Organization estimated 100 million cases of HAV infection worldwide, resulting in 15,000–30,000 deaths annually. In middle‐ and low‐income countries with poor hygiene and sanitation standards, individuals are exposed to HAV at an early age, with a prevalence exceeding 90% in children under 10 years old, leading to a high proportion of immune adults [2]. HAV infection presents with a broad clinical spectrum, ranging from mild cases without complications to severe cases with acute liver failure, gangrene, shock, and death. Common symptoms include fever, malaise, diarrhea, nausea, abdominal pain, and jaundice; however, these are often nonspecific [3]. HAV can also cause extrahepatic complications, with acute acalculous cholecystitis (AAC) being a rare finding [4]. Other uncommon conditions associated with HAV infection include skin rashes, pancreatitis, acute kidney injury, hematological disorders, Guillain–Barré syndrome, and myocarditis [5]. In recent years, HAV‐associated AAC has been reported mainly in children and rarely in adults from developing countries, with only a few cases documented to date [6–9]. In this case report, we present a 31‐year‐old woman who developed HAV‐associated AAC, and we discuss the patient’s management until resolution. While AAC associated with HAV is more commonly described in pediatric populations, adult cases are rare and often underreported. Chang et al. [8] described a severe adult case requiring surgical intervention, whereas Kaya et al. [9] reported a spontaneously resolved presentation. These contrasting reports highlight the variability in clinical progression and support the need for detailed documentation of adult cases.

## 2. Case Report

A 31‐year‐old woman from Lima in Peru, presented with a 5‐day history of progressive epigastric pain, fever (38.5°C), early satiety, nausea, and two episodes of vomiting. Physical examination revealed a positive Murphy’s sign and hepatomegaly. Initial imaging and laboratory results raised suspicion of acute cholecystitis, prompting further evaluation prior to surgical intervention.

Ultrasound revealed reactive gallbladder changes and mild bile duct dilation (Figure 1(a)), while follow‐up imaging showed significant mural thickening (Figure 1(b)). MRCP confirmed AAC and excluded biliary obstruction (Figure 2). Liver enzymes and bilirubin levels demonstrated a cholestatic pattern (Table 1).

Figure 1Upper abdominal ultrasound (a). Size: 57 × 21 mm. Wall thickness: 2.9 mm. No gallstones observed. Dense content is present inside, suggesting reactive changes. (b) Collapsed gallbladder with an increased volume compared with the previous measurement (63 × 39.8 mm), with thickened walls up to 20 mm and signs of hyperemia, suggesting a reactive process related to the underlying pathology. Free perivesicular fluid of 1.5 cc is observed. (c) Collapsed gallbladder measuring 48 × 15.9 mm. Wall thickness: 4.1 mm. Anechoic content is present. Laminar free perivesicular fluid is observed.

**Figure:**
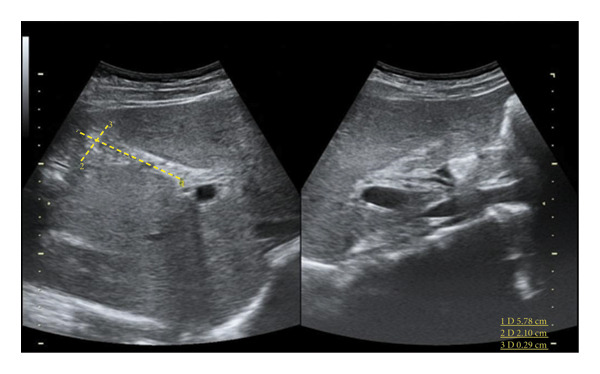
(a)

**Figure:**
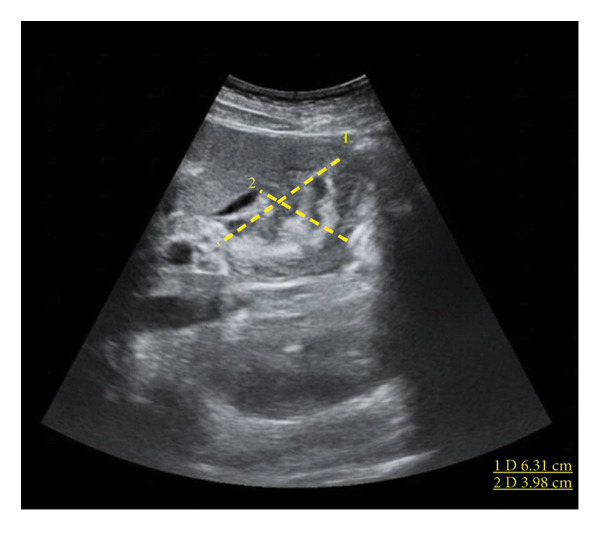
(b)

**Figure:**
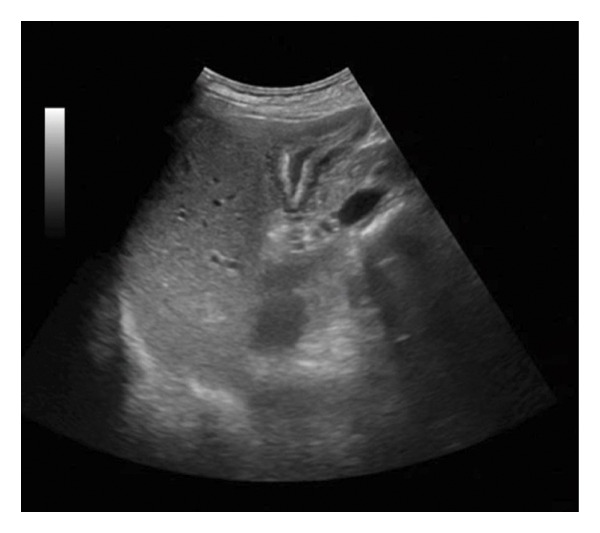
(c)

Figure 2T2 sequences (a), T2 Fat‐Sat (b), diffusion (c), and ADC (d) acquired in the coronal plane at the hepatic hilum and axial plane at the gallbladder level, showing periportal edema (^∗^), diffuse mural thickening of the gallbladder (°), molecular restriction of the gallbladder content (´), and laminar perivesicular fluid (<).

**Figure:**
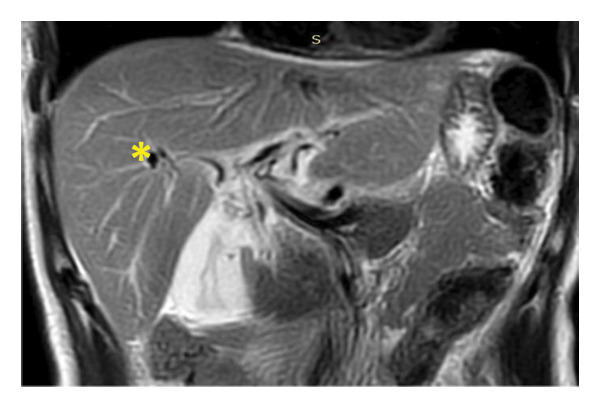
(a)

**Figure:**
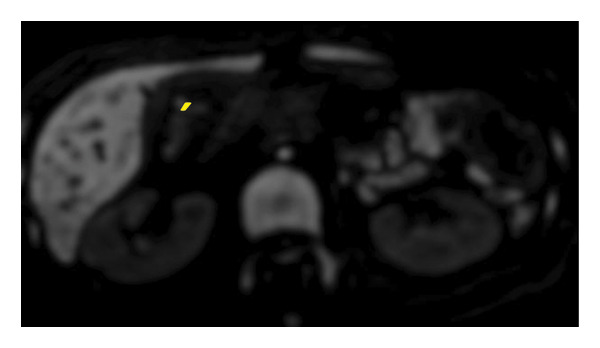
(b)

**Figure:**
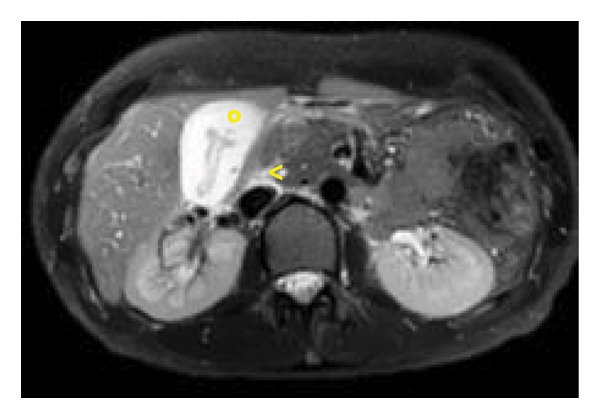
(c)

**Figure:**
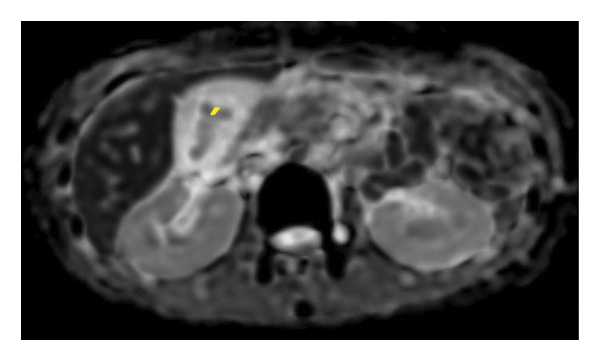
(d)

**Table 1:** Liver function panel.

	Day 0	Day 2	Day 3	Day 5	Day 7	Day 21
Enzymes						
AST(U/L)	3636.2	4757.4	2639.3	953.6	234.9	41.5
ALT (U/L)	3243	3649.4	3042.3	1606	911.1	36.5
Alkaline phosphatase (U/L)	540	—	—	320	309	77
GGT (U/L)	356	—	—	150	167	8
Bilirubins						
Total bilirubin (mg/dL)	—	6.03	7.61	9.97	12.36	0.2
Direct bilirubin (mg/dL)	—	5.97	7.26	9.74	11.74	0.11
Indirect bilirubin (mg/dL)	—	0.06	0.35	0.23	0.62	0.09

*Note:* U/L, units per liter; mg/dL, milligrams per deciliter.

Abbreviations: ALT, alanine aminotransferase; AST, aspartate aminotransferase; GGT, gamma‐glutamyl transferase.

The case was managed by the internal medicine team. Serological tests confirmed acute Hepatitis A infection, ruling out autoimmune or other viral causes (Table 2), leading to the definitive diagnosis of AAC secondary to Hepatitis A. On targeted anamnesis, the patient denied consuming food outside her home or drinking water from stagnant sources. An infectious disease consultation recommended antibiotic therapy due to hepatic involvement and the severity of the cholecystitis; however, a conservative approach was chosen, given the self‐limiting nature of the viral infection.

**Table 2:** Virological markers.

Serological test	Result
Anti‐VAH IgM	Positive
Anti‐VAH IgG	Negative
Epstein Barr IgM	Negative
Citomegalovirus IgM	Negative
HBsAg	Negative
Anti‐HCV	Negative
Anti‐HEV	Negative
SARS‐CoV‐2 PCR	Negative
Autoinmunes	
Anti‐Ro, Anti‐La, Anti‐Sm, DNA‐ds, ANA	Negative

*Note:* Anti‐HAV, antibody against hepatitis A virus; HBsAg, hepatitis B surface antigen; Anti‐HCV, antibody against hepatitis C virus; Anti‐HEV, antibody against hepatitis E virus.

Liver and inflammatory markers began to decrease within 48 h of admission. The treatment consisted of intravenous hydration during the first 24 h, followed by tramadol 50 mg, metoclopramide 10 mg, and omeprazole 40 mg. During hospitalization, the patient showed favorable clinical progress, with pain reduction, progressive oral tolerance, and a decrease in gallbladder inflammation, which normalized by Day 7 (Figure 1(c)). During follow‐up visits, the patient remained in good general condition, and laboratory values returned to normal without any abnormalities. To the best of our knowledge, the patient did not experience any complications or sequelae following discharge.

## 3. Discussion

Each year, approximately 200,000 cases of acute cholecystitis are diagnosed; however, AAC accounts for less than 5%. This condition is characterized by acute inflammation of the gallbladder in the absence of gallstones. Its etiology is complex and multifactorial, with possible mechanisms including direct invasion of gallbladder epithelial cells, vascular inflammation, and bile duct obstruction. These alterations may lead to biliary stasis resulting from reduced motility and inflammatory edema of the gallbladder wall may contribute to functional obstruction and secondary inflammation, ischemia due to microvascular compromise has been suggested, particularly in the setting of systemic inflammatory response and hepatic dysfunction, necrosis, or even spontaneous bacterial peritonitis, particularly in critically ill and immunosuppressed patients. In addition, cell‐mediated immunologic has also been proposed, in which circulating immune complexes and local cytokine release trigger endothelial injury and mucosal inflammation [1, 5]. Clinically, AAC can be confused with calculous cholecystitis, as they share symptoms such as nausea, vomiting, colicky abdominal pain, jaundice, and a positive Murphy’s sign. Imaging studies are crucial for diagnosis, with abdominal ultrasound being the preferred tool due to its accessibility, low cost, and ease of use. This method allows the identification of signs such as gallbladder wall thickening (> 3.5 mm), pericholecystic fluid, intramural edema, or hypertrophic mucosa. Additionally, ultrasound offers a specificity of 97.8% and an accuracy of 96.1%, although its effectiveness depends on the operator. Many of these alterations occur in critically ill patients with underlying infections. Laboratory tests for infection, including blood counts, C‐reactive protein (CRP), renal function, and cholestasis markers, are not always altered and are, therefore, nonspecific. A thorough history and complementary tests remain essential for diagnosis, while computed tomography and scintigraphy can be useful for differential diagnosis [10]. In our patient, we found an inflamed gallbladder (Figure 1(c)), accompanied by leukocytosis and an altered hepatic profile with a cholestatic pattern, showing significantly elevated GGT and alkaline phosphatase levels (Table 1). Hepatitis A infection is common in children but rare in adults, usually presenting with mild symptoms. However, it should be suspected when transaminase levels are more than three times the normal range without another apparent cause, as its clinical presentation is often nonspecific [11]. The treatment of AAC focuses on symptom control. The literature mentions that antibiotics have been proposed as a therapeutic option against microorganisms that colonize bile acids. However, intravenous supportive therapy may be sufficient in most cases where the systemic condition is self‐limiting [12, 13]. When medical treatment fails, surgery is rarely required, only in cases of persistent abdominal pain or perforation. In this case, antibiotics were not administered, as the acalculous cholecystitis was associated with self‐limiting Hepatitis A. A conservative treatment approach based on supportive therapy and continuous evaluation was chosen, which proved effective in controlling symptoms and resolving gallbladder inflammation, as evidenced by the progressive reduction of gallbladder size in subsequent ultrasound evaluations. While many adult cases resolve with supportive care, others may progress to complications requiring antibiotics or surgery. Close monitoring and individualized clinical judgment are essential. Comparative reports, such as Chang et al. [8] requiring surgery versus Kaya et al. [9] resolving spontaneously, illustrate the clinical variability. Table 3 summarizes previously reported adult cases of HAV‐associated AAC, highlighting variations in clinical presentation, management strategies, and outcomes.

**Table 3:** Summary of reported adult cases of hepatitis A virus–associated acute acalculous cholecystitis.

Author (year)	Age (year)	Presentation	Treatment	Outcome
Kaya et al. (2013) [9]	24	Jaundice, RUQ pain; ↑ AST/ALT	Supportive care	Full recovery
Chang et al. (2023) [8]	35	Fever, RUQ pain, liver failure; ↑↑↑ AST/ALT	Surgery + antibiotics	Recovery post‐surgery
Present case	31	Epigastric pain, fever; AST > 3600	Conservative treatment	Full recovery

This report presents a single clinical case, which inherently limits the generalizability of its findings. Clinical outcomes in similar cases may differ depending on patient comorbidities, immune status, and the timing of diagnosis and intervention. Further observational studies and larger case series are necessary to better characterize the clinical spectrum and to establish evidence‐based management strategies for HAV‐associated AAC in adults.

## 4. Conclusions

AAC secondary to hepatitis A should be considered in patients presenting with nonspecific biliary symptoms, gallbladder distension, and hepatic biochemical abnormalities. Early use of imaging and serologic testing is essential for accurate diagnosis and to prevent unnecessary surgical interventions. This case underscores that conservative management with supportive therapy can be effective in carefully selected adult patients when the underlying etiology is viral and self‐limiting. Recognizing HAV as a potential cause of AAC broadens clinical awareness and supports a more judicious approach to the use of antibiotics and surgery, ultimately improving patient outcomes.

## Consent

The patient provided written informed consent for the publication of this case report.

## Conflicts of Interest

The authors declare no conflicts of interest.

## Funding

No funding was received for this manuscript.

## Data Availability

The data that support the findings of this study are available from the corresponding author upon reasonable request.
